# Wearable multimodal sensing for quantifying the cardiovascular autonomic effects of levodopa in parkinsonism

**DOI:** 10.3389/fnetp.2025.1543838

**Published:** 2025-04-24

**Authors:** John A. Berkebile, Omer T. Inan, Paul A. Beach

**Affiliations:** ^1^ School of Electrical and Computer Engineering, Georgia Institute of Technology, Atlanta, GA, United States; ^2^ Wallace H. Coulter Department of Biomedical Engineering, Georgia Institute of Technology, Atlanta, GA, United States; ^3^ Department of Neurology, Emory University School of Medicine, Atlanta, GA, United States

**Keywords:** wearable sensing, Parkinson’s disease, autonomic dysfunction, cardiovascular monitoring, multiple system atrophy, parkinsonism, levodopa, ambulatory monitoring

## Abstract

Levodopa is the most common therapy to reduce motor symptoms of parkinsonism. However, levodopa has potential to exacerbate cardiovascular autonomic (CVA) dysfunction that may co-occur in patients. Heart rate variability (HRV) is the most common method for assessing CVA function, but broader monitoring of CVA function and levodopa effects is typically limited to clinical settings and symptom reporting, which fail to capture its holistic nature. In this study, we evaluated the feasibility of a multimodal wearable chest patch for monitoring changes in CVA function during clinical and 24-h ambulatory (at home) conditions in 14 patients: 11 with Parkinson’s disease (PD) and 3 with multiple system atrophy (MSA). In-clinic data were analyzed to examine the effects of orally administered levodopa on CVA function using a pre (OFF) and 60-min (ON) post-exposure protocol. Wearable-derived physiological markers related to the electrical and mechanical activity of the heart alongside vascular function were extracted. Pre-ejection period (PEP) and ratio of PEP to left ventricular ejection time index (LVETi) increased significantly (p < 0.05) following levodopa, indicating a decrease in cardiac contractility. We further explored dose-response relationships and how CVA responses differed between participants with orthostatic hypotension (OH) from those without OH. Heart rate variability, specifically root-mean-square-of-successive-differences (RMSSD), following levodopa decreased significantly more in participants with OH (n = 7) compared to those without (no-OH, n = 7). The results suggest that the wearable patch’s measures are sensitive to CVA dynamics and provide exploratory insights into levodopa’s potential role in inducing a negative inotropic effect and exacerbating CVA dysfunction. This work encourages further evaluation of these wearable-derived physiomarkers for quantifying CVA and informing individualized care of individuals with parkinsonism.

## 1 Introduction

Parkinson’s disease (PD) and parkinsonian multiple system atrophy (MSA) are alpha-synucleinopathies ( α SNA) primarily characterized by motor impairments involving rigidity, bradykinesia, and resting tremor. Levodopa is the gold standard therapeutic for combating motor symptoms in α SNA (Yahr et al., 1969; Olanow et al., 2004; Sprenger and Poewe, 2013), but its effects on cardiovascular autonomic (CVA) function can limit or preclude its use for some patients. Impaired CVA function, quantified with measures of heart rate variability (HRV), have long been reported in PD independent of levodopa treatment (Kallio et al., 2000; Bouhaddi et al., 2004). Further, these impairments appear to worsen with disease severity (Devos et al., 2003; Mesec et al., 1993). Clinically, levodopa’s potential hypotensive effect (Calne et al., 1970; Goldberg and Whitsett, 1971; Kujawa et al., 2000; Noack et al., 2014; Cani et al., 2024) is the most notable concern as it can cause or exacerbate orthostatic hypotension (OH), a hallmark manifestation of autonomic failure in PD and MSA (Senard et al., 1992; Goldstein et al., 2005; Freeman, 2008).

The reported cardiovascular effects of levodopa vary widely as studies often differ in route of administration, dosage, type and timing of physiological measurements, and participant characteristics. When administered in large doses, levodopa can produce a sustained positive inotropic effect in individuals with PD and heart failure (Whitsett and Goldberg, 1972; Rajfer et al., 1984); however, in studies of parkinsonian individuals, typical therapeutic amounts for motor parkinsonism observed a negative inotropic effect (Wolf et al., 2006; Noack et al., 2014), with reductions in stroke volume and blood pressure (BP) (Bouhaddi et al., 2004; Liu et al., 2023; Cani et al., 2024; Earl et al., 2024). Notably, these cardiovascular effects have primarily been assessed with traditional bench-top sensors, confining this form of physiological monitoring of levodopa responses to controlled clinical settings. In addition to its hemodynamic effects, some studies have reported that levodopa induces alterations in short-term HRV measures (Sriranjini et al., 2011; Ruonala et al., 2015). Given the complexity of levodopa’s influence on both hemodynamic and autonomic function, wearable sensing technologies capable of continuously extracting markers of CVA function beyond HRV alone could enhance the characterization of levodopa’s effects and facilitate measurements in a broader range of environments.

Outside of monitoring motor symptoms in parkinsonisms (Patel et al., 2009; Ossig et al., 2016; Heijmans et al., 2019; Kamo et al., 2024), the use of wearable technology in α SNA remains limited. In recent work, wearable sensors have been used to measure non-motor markers to detect and predict a patient’s onset of wearing off of levodopa effectiveness (Arasteh et al., 2023; Barrachina-Fernández et al., 2023). The wristwatch devices employed in these studies primarily focus on measuring electrodermal activity (EDA), due to its association with sympathetic activity (Posada-Quintero et al., 2016). While effective for detecting wearing-off of levodopa, EDA lacks sensitivity to the cardiovascular changes that levodopa evokes, which are clinically significant due to the drug’s potential hypotensive effects. Approaches that leverage continuous wearable sensing of CVA markers, such as those linked to hemodynamic function, may provide a more effective method for the detection of OH.

To address these gaps, we propose the use of a multimodal chest patch that enables the monitoring of markers closely related to CVA function, such as HRV, systolic timing intervals, and vascular reactivity. The wearable patch measures raw physiological waveforms including electrocardiogram (ECG), seismocardiogram (SCG) and photoplethysmogram (PPG) signals (Chan et al., 2021) from which several indices of CVA can be extracted on a beat-by-beat basis. The pre-ejection period (PEP) and left ventricular ejection time (LVET), both of which can be derived from the combination of ECG and SCG signals, are strongly associated with changes in beta-adrenergic sympathetic activity, cardiac contractility, and, consequently, stroke volume (Lewis et al., 1977; Kelsey, 2012). Notably, SCG has not been used, to our knowledge, to characterize CVA responses to levodopa in individuals with parkinsonism, despite its demonstrated efficacy in other contexts (Inan et al., 2015). We hypothesize that sensing both cardiomechanical and vascular activity, measured via PPG signals, will provide a more comprehensive understanding of levodopa’s hypotensive effects and provide early indications suggesting their underlying mechanisms.

**FIGURE 1:**
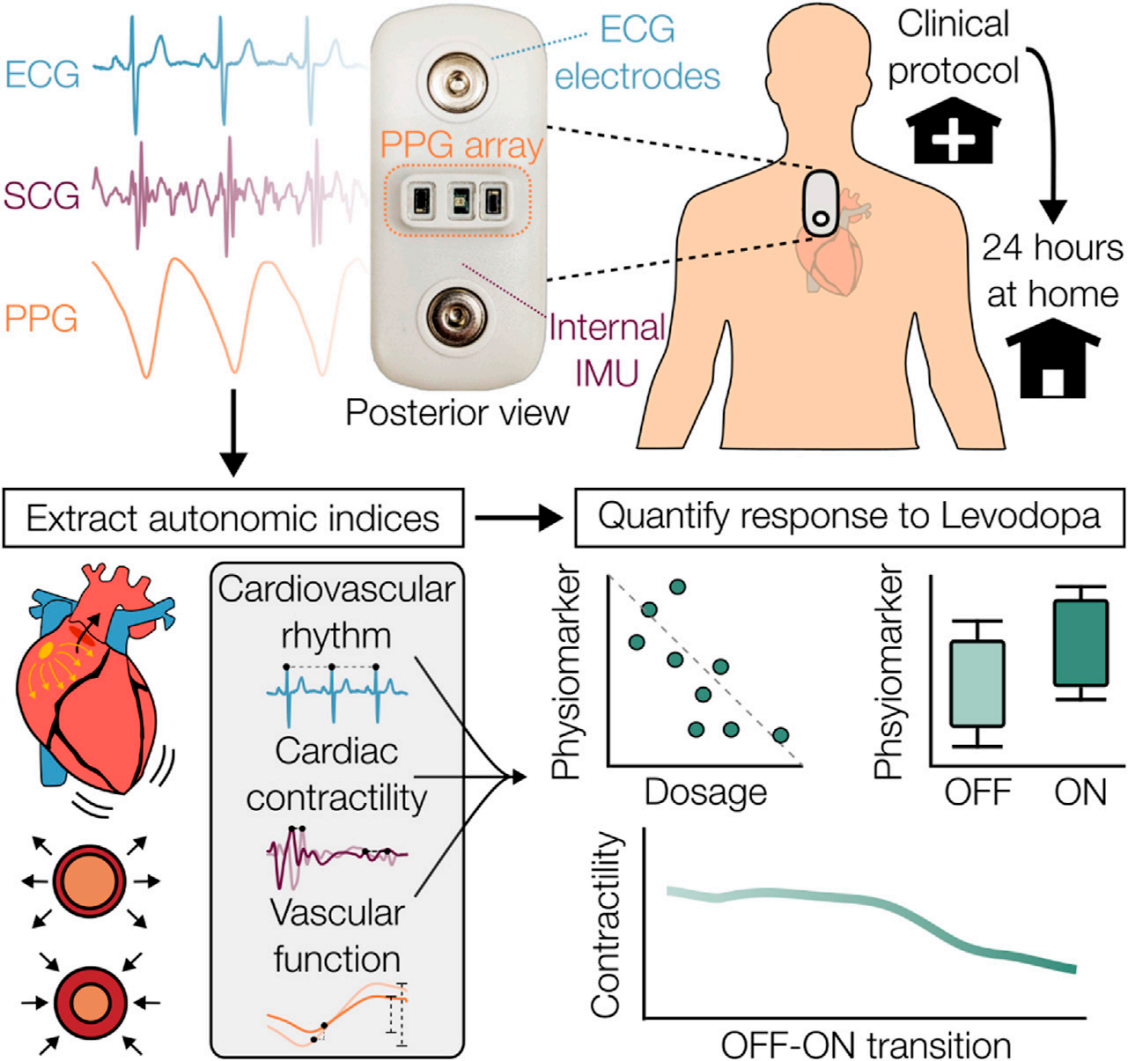
Overview of wearable sensing system and aims of this study. A wearable chest patch that measures electrocardiogram (ECG), seismocardiogram (SCG), and photoplethysmogram (PPG) signals was used in a population of Parkinson’s disease and multiple system atrophy during a clinical protocol and 24-h ambulatory study at home. Physiological markers related to cardiovascular autonomic function were extracted from the patch’s signals and were used to characterize the response to levodopa.

The aim of this study is to assess the feasibility of using wearable multimodal sensing to monitor levodopa-induced changes in CVA function and to investigate the potential of wearable-derived physiological markers for capturing these effects. The conceptual design of this work is illustrated in Figure 1. We collected data with a wearable chest patch from n = 14 participants with PD (n = 11) and MSA (n = 3) during a clinical protocol, where the participants received their typical dosage of dopaminergic medication, and during a 24-h period at home. We examined the patch’s markers of CVA function before (OFF) and after levodopa administration (ON), for the first time, in both clinic and at-home settings. We additionally explored how the magnitude of the CVA responses captured by the patch were related to the administered dosage, and how they differed between participants with OH from those without OH. As the use of wearables for CVA monitoring in parkinsonian populations has been scarce, this work advances the state of wearable research and provides complementary insight into the acute CVA effects of levodopa, providing a framework to further explore these findings.

## 2 Materials and methods

### 2.1 Study protocol

**FIGURE 2:**
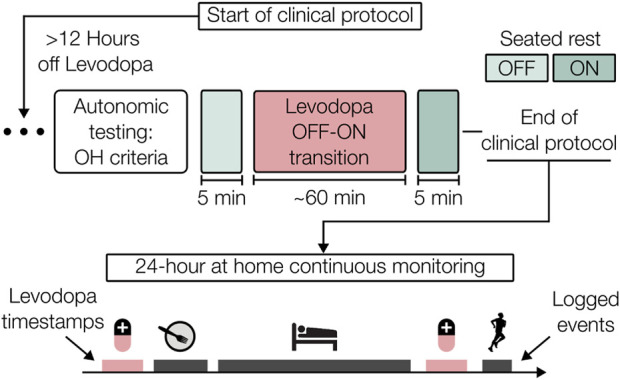
Clinical and at-home protocol overview. After a 12-h washout period, participants underwent autonomic testing followed by administration of their typical oral levodopa medication. The OFF-ON response was measured and then a 24-h period of continuous at-home monitoring was carried out. Participant diaries were used to provide event timestamps, including administration of medications.

The experimental protocol, summarized in Figure 2, was conducted under approval by the institutional review boards of both the Georgia Institute of Technology (#H21492) and Emory University School of Medicine (#00003055). Participants with mild to moderate idiopathic PD (Hoehn and Yahr, stages 1–3 (Hoehn and Yahr, 1967)) or the parkinsonian variant of MSA were enrolled. Participants with a medical history of cardiac diseases, arrhythmias, or other neurologic diseases beyond PD or MSA were excluded.

The protocol began early in the morning after a minimum of 12 h abstinence from dopaminergic medications, vasoactive medications, caffeine, and other neurodepressants and neurostimulants, including medications to combat OH, to mitigate the potential cardiovascular influences of these substances. OFF-state motor symptom severity was examined via Part 3 motor sub-scale of the Movement Disorders Society Unified Parkinson’s Disease Rating Scale (MDS-UPDRS III) (Goetz et al., 2008) by a movement disorders trained neurologist. After the wearable patch and additional sensors were attached, data were acquired from periods of quiet rest and during autonomic function testing. An active standing challenge was performed to identify OH according to the census criteria, which specifies a sustained drop of at least 20 mmHg systolic BP (SBP) or 10 mmHg diastolic BP (DBP) within 3 min of upright posture (Freeman et al., 2011). Reference BP values were acquired continuously by either a ccNexfin system (Edwards Lifesciences, Irvine, CA, USA) or a CNAP monitor (CNSystems, Austria), with a VS 9 brachial cuffed BP monitor (Mindray, Shenzhen, China) to confirm the presence of OH.

Following the completion of these maneuvers in the OFF-medication state, the participant’s typical levodopa equivalent dosage (LED) was administered orally. Sensors other than the wearable patch were disconnected and a battery of symptom related questionnaires and assessments were completed. After approximately 60 min had passed, a time imposed to allow for the dopaminergic medication to take full effect (Noack et al., 2014), motor symptoms were reassessed (ON-state).

Participants were then given the wearable patch to wear for the next 24 h with an autonomic diary to log events relevant to autonomic function such as meals, administration of medications, sleep, and symptoms suggestive of OH (e.g., dizziness, light-headedness). Participants were instructed to carry out their normal activities of daily living and partake of their normal medications while wearing the patch. The patch was removed for brief periods to replace the ECG electrodes, if needed, or while bathing. Upon return of the wearable patch by the participant, waveform data were extracted from the onboard secure digital (SD) card and the logged autonomic diary events were converted to digital timestamps to contextualize the data.

### 2.2 Wearable multimodal sensor

A multimodal wearable patch, shown in Figure 1, was attached to the mid-sternum, roughly 2 cm below the suprasternal notch, to capture physiological waveforms pertinent to CVA activity. The patch was adhered to the chest with a pair of standard 3M-2670 Ag/AgCl gel electrodes (3M, St. Paul, MN, USA). This device acquires single-lead ECG, two sets of multiwavelength—infrared (IR), red, and green—PPGs, and triaxial acceleration from which the SCG is derived. The ECG, PPG, and SCG signals are sampled at 500, 67, and 1,000 Hz, respectively. The wearable patch’s hardware remains unchanged from prior studies (Chan et al., 2021; Berkebile et al., 2023). To briefly summarize: the ECG signal is measured from the ADS1292 (Texas Instruments, Dallas, TX, USA) analog front-end (AFE), the ADXL355 (Analog Devices, Norwood, MA, United States) digital low-noise accelerometer captures the triaxial SCG signal, and a combination of the Maxim 86,170 (Maxim Integrated, San Jose, California, United States) AFE, SFH 7016 (OSRAM, Munich, Germany) light emitting diodes, and VEMD 8080 (Vishay Semiconductors, Heilbronn, Baden Württemberg, Germany) photodiodes enable the extraction of reflectance-based PPG. Similar versions of this hardware have been deployed and validated in studies investigating heart failure (Inan et al., 2018), stroke volume monitoring (Ganti et al., 2022), and stress in participants with myocardial infarction (Nawar et al., 2023).

### 2.3 Segmenting OFF and ON periods

In accordance with prior work (Sriranjini et al., 2011; Noack et al., 2014; Ruonala et al., 2015), we analyzed periods prior to levodopa administration (OFF) and an hour after taking levodopa (ON) to assess its effects on CVA function. Raw waveforms measured by the wearable patch during 5-min periods of seated rest were extracted from both OFF and ON states during the clinical protocol. The separation between these two periods was as close to 60 min as possible, but was dependent on participant behavior, e.g., bathroom break or delayed completion of assessments. We also analyzed at-home levodopa administrations to gain insight into CVA responses outside of controlled clinical environments. Levodopa administration times were logged in the participant diaries during the 24-h at-home portion of the study. These times were referenced alongside the wearable patch’s accelerometer to identify periods of inactivity similar to those employed in the clinical portion (Godfrey et al., 2011). 5-min periods were selected as close as possible to the administration time and 60 min following administration. To ensure consistency, these periods were further constrained to ensure that posture was uniform across the OFF and ON periods, with minimal activity during both the 5-min interval and immediately preceding the period.

### 2.4 Signal processing and heartbeat segmentation

**FIGURE 3:**
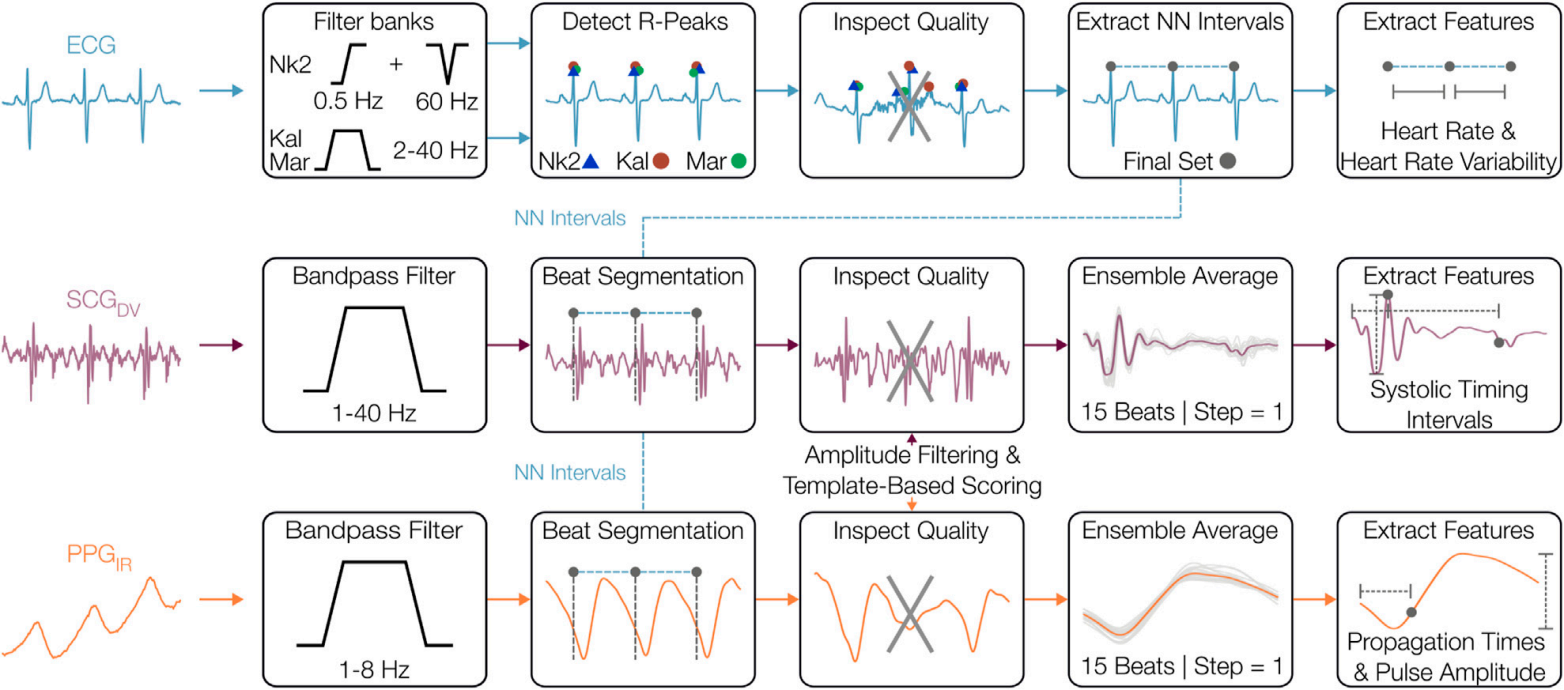
Signal preprocessing and physiomarker extraction. The electrocardiogram (ECG), seismocardiogram (SCG), and photoplethysmogram (PPG) signals were initially filtered to their respective frequency bands where cardiovascular information is most prominent. From the ECG, R-peaks were located and used to extract normal-normal (NN) intervals, from which the other signals were segmented on a beat-by-beat basis. The quality of these beats was assessed using template distance metrics and amplitude outlier removal. The SCG and PPG beats were then ensemble-averaged before features were extracted from each heartbeat.

The raw wearable patch signals acquired during the OFF and ON states were preprocessed in Python (3.10.14) unless otherwise noted. The signal processing and physiological marker (physiomarker) extraction pipeline is shown in Figure 3 and largely follows that of our prior work (Berkebile et al., 2025). Only the dorsoventral (DV) component of the SCG and the IR wavelength PPG were employed in this work to capture cardiomechanical and vascular activity, respectively. Firstly, the ECG, SCG, and PPG waveforms were uniformly resampled to 500 Hz. The SCG and PPG signals were bandpass filtered to 1–40 Hz and 1–8 Hz, respectively. The ECG was passed through multiple filter banks as three R-peak detectors were used from the Neurokit2 (NK) toolbox: the NK method, Kalidas (Kal) method, and Martinez (Mar) method (Makowski et al., 2021; Kalidas and Tamil, 2017; Martinez et al., 2004). The final set of R-peaks was obtained by fusing the three individual R-peak sets, with the NK method serving as the reference. Only the R-peaks that multiple detectors successfully located within a lenient tolerance of 150 ms were reserved for further use. This fusion improved the reliability of R-peak detection for participants where individual detectors were less effective on the single-lead ECG waveform. The fused set of R-peaks formed R-R intervals which were filtered based on physiological implausibility for the given conditions (outside of 40–180 bpm) and flagged for outliers. Outliers were designated using sliding windows (30-beat, 90% overlap), flagging values ± 5 median absolute deviation (MAD) away from the window’s median. This final set of R-R intervals formed the normal-to-normal (NN) intervals, which were manually inspected for accuracy and were used for extracting HRV parameters.

The filtered SCG and PPG waveforms were segmented into heartbeats using the R-peaks with a fixed duration of 600 ms, which captures the salient systolic cardiac events and simplifies the signal processing (Shandhi et al., 2021). These SCG and PPG beats underwent signal quality assessment including amplitude-based outlier removal and local morphological similarity scoring. First, outlier SCG and PPG beats were removed using both global and sliding windows (30 beats, step = 1) or beat amplitudes, with beats flagged if the amplitude was ± 5 MAD away from the respective global and window medians (Gazi et al., 2021). Next, morphological outliers were removed by generating SCG and PPG templates (30-beat windows, no overlap) and assessing the distance each beat in the window to the template. Dynamic time feature matching (DTFM) (Zia et al., 2019) and dynamic time warping (DTW) (Giorgino, 2009; Li and Clifford, 2012) metrics were used as the distance metrics for the SCG and PPG assessments, respectively, to harness the expected similarities in the physiological structure of beat-segmented waveforms. Specifically, DTFM expands upon DTW, which estimates the distance between two signals allowing for expansion or compression in time, by additionally imposing constraints regarding the matching of time-domain features on the SCG signal. The thresholds imposed on the distance metrics were heuristically determined, with a value of 0.4 for PPG signals and 0.3 for SCG signals, to balance the extent of data removal with the reliability of morphological features. Once outlier SCG and PPG beats were excluded, the final set of beats were ensemble-averaged (15 beats, step = 1) to mitigate uncorrelated noise.

### 2.5 Physiomarker extraction

After the preprocessing stage, physiological markers (physiomarkers) related to CVA function were extracted from known landmarks found in ECG, SCG, and PPG beats. For ECG-related features, an HR time-series was computed from NN intervals and was further used in the computation of several time and frequency indices of HRV using the NK library. HRV parameters were computed from 5-min windows. After replacing missing values in the NN interval time-series with piecewise cubic Hermite interpolating polynomial (pchip) interpolation (Morelli et al., 2019; Benchekroun et al., 2023), time-domain parameters including root mean square of successive differences (RMSSD) and standard deviation of NN intervals (SDNN) were computed. The NN intervals were first resampled to 8 Hz before computing frequency metrics (Clifford and Tarassenko, 2005; Singh et al., 2004). Using NK’s implementation of the Welch method, the power in the LF band [0.04–0.15 Hz] and HF band [0.15–0.4 Hz] were derived, as well as the LF/HF ratio. The length of the analysis window was computed following the NK default implementation, which ensures at least two cycles of the lowest detectable frequency (0.013 Hz) are captured in each analysis window. An overlap of 50% was used to improve the robustness of spectral estimates. These HRV parameters were selected as some of the most commonly used to quantify levodopa responses in relevant work (Sriranjini et al., 2011).

Physiomarkers related to cardiac contractility and vasomotor reactivity were obtained from the SCG and PPG signals. PEP and HR-corrected LVET (LVETi) values, as well as their ratio PEP/LVETi, were derived from the aortic valve opening (AO) and the aortic valve closing (AC) fiducial points identified on the SCG (Lewis et al., 1977; Zia et al., 2019). Due to intersubject variability and longitudinal differences in SCG morphologies, multiple prominent peaks and valleys of the SCG waveform that strongly covary were tracked as candidate fiducial points. The median of these candidates was taken to mitigate errors in the PEP and LVET estimates that could occur in single candidate tracking. Note that these PEP and LVET estimates may be slightly offset from the true PEP and LVET, due to the complexity of SCG morphology, but reliably track longitudinal changes in these timings (Lin et al., 2021). The maximum and minimum points in the AO complex were used to compute the SCG amplitude ( ${\text{SCG}}_{\text{amp}}$ ). PPG physiomarkers also consisted of amplitude and timing features. The pulse amplitude ( ${\text{PPG}}_{\text{amp}}$ ) was computed for each beat as the difference in the maximum and minimum amplitude. Pulse arrival time (PAT) was extracted as the time between the R-peak and foot of the PPG, the latter of which was identified as the median of multiple metrics including the maximum, minimum, maximum of first derivative, maximum of second derivative, 20% of pulse height, and intersecting tangents (Mukkamala et al., 2015; Hemon and Phillips, 2016). Fusing the PEP and PAT estimates formed the PTT, which is the difference between the PAT and the PEP.

### 2.6 Statistical analysis of levodopa responses

The wearable-based features extracted during the 5-min seated periods in both OFF and ON states were averaged for each subject and then grouped by medication state for analysis. The features were subsequently compared to identify any levodopa-induced change in resting physiology, akin to comparisons made in (Noack et al., 2014). While acknowledging the pathophysiological differences between PD and MSA regarding the origins of autonomic failure (Iodice et al., 2011), patients with PD and MSA were analyzed together given that levodopa-induced worsening of orthostatic hypotension is a commonly observed effect in both conditions (Coon and Ahlskog, 2021). The comparisons included all 13 wearable patch features. Prior to comparisons between groups, the groups were tested for normality with the Shapiro-Wilk test and equality of variance via Levene’s test. If either normality or equal variance was rejected, a two-sided Wilcoxon signed-rank test was performed; otherwise, a two-sided paired t-test was performed. Cohen’s effect size (d) for paired comparisons was computed. Separately, the physiomarkers acquired in the OFF-ON levodopa transitions detected at home required linear mixed effect models to account for repeated measurements, as most participants took levodopa multiple times over the 24-h monitoring period. The effect of state (OFF vs. ON) was examined on each physiomarker, with random intercepts fit for each participant.

Due to participants’ typical LED being taken, we investigated the relationship of the magnitude of physiomarker changes from OFF to ON with the LED that was administered. These correlations used Spearman’s ρ in instances where the Shapiro-Wilk test indicated that the data were not normally distributed; otherwise, Pearson’s r was used.

The potential hypotensive effects of levodopa are of primary interest in people with α SNA, where known or latent autonomic dysfunction may be worsened by levodopa-induced changes in CVA function. Accordingly, we grouped the participants into OH and no-OH groups and compared the magnitude of the OFF-ON changes between groups as measured in the clinic. Groupwise differences in CVA reactivity were assessed with two-sided independent t-tests. If the assumption of normality was not met, Mann-Whitney U tests were used instead. Cohen’s d was computed for each comparison. A similar analysis was carried out at home, again using linear mixed effect models due to repeated measures. The effect of group (OH vs. no-OH) was examined for each physiomarker.

In alignment with the aims of this study, which is focused on whether levodopa affects the patch’s multimodal physiomarkers in addition to the commonly employed HRV markers, Benjamini–Hochberg corrections were applied to control the false discovery rate across related physiological parameters (Benjamini and Hochberg, 1995). Specifically, HR and HRV parameters were grouped together, representing the most studied markers of autonomic regulation. Meanwhile, the cardiomechanical and vasomotor-related physiomarkers from SCG and PPG signals were analyzed as a separate group. P values are accordingly corrected for each set of analyses. Given the exploratory nature of this study, we report both adjusted ( ${\text{p}}_{\text{adj}}$ ) and unadjusted p-values (p) and clearly denote results as significant before or after correction. All the above statistical tests were handled in this manner.

## 3 Results

### 3.1 Dataset characteristics

Wearable sensor data were collected from n = 14 participants, 11 with PD and 3 with MSA, for the clinical portion. OH was confirmed through autonomic testing in n = 7 participants, 2 with MSA and 5 with PD. The clinicodemographics, motor symptom assessments, and levodopa dosages are detailed further in Table 1. Average supine, seated, and standing heart rates and BPs, measured during the autonomic testing are reported in Supplementary Table I. Motor testing in the ON-state revealed significant reductions in the MDS-UPDRS III score (p < 0.001, t = 7.978, d = 2.213) and Hoehn-Yahr scale (p = 0.025, U = 0.0, d = 0.745) compared to OFF-state, further recorded in Supplementary Table II. The time between the clinical OFF and ON periods used in the physiomarker analysis was 61.4  ± 7.9 (mean ± SD) minutes. Following the clinical testing, n = 13 participants continued wearing the chest patch for 24 h of at-home monitoring. OFF-ON levodopa-related transitions viable for subsequent analyses were identified for 12 of the 13 participants, as one participant did not take levodopa during the entire at-home period. A total of N = 42 levodopa responses were captured from the at-home portion, but 5 were excluded due to differences in posture for the OFF-ON periods. An additional response was rejected due to poor SCG signal quality; thus, a final count of N = 36 levodopa responses were analyzed, with 3 ± 1 OFF-ON transitions per participant. The time between the OFF and ON periods used in the at-home analysis was 59.6 ± 5.1 min. Supplementary Table III details concomitant blood pressure-related medications taken by participants at home.

**TABLE 1:** Cohort characteristics.

Parameter	Overall N = 14^a^	MSA N = 3^a^	PD N = 11^a^	p-val^b^
Sex (female)	5	2	3	
OH (n)	7	2	5	
Age (yrs)	68.5 (9.8)	65.3 (3.8)	69.4 (10.9)	0.31
Disease duration (yrs)	3.8 (3.5)	0.6 (0.1)	4.7 (3.4)	0.01
MDS-UPDRS III (OFF)	31.9 (10.2)	32.7 (8.7)	31.6 (11.0)	>0.99
MDS-UPDRS III (ON)	20.1 (9.2)	25.3 (11.5)	18.7 (8.6)	0.44
Hoehn-Yahr (OFF)	2.1 (0.5)	2.3 (0.6)	2.1 (0.5)	0.56
Hoehn-Yahr (ON)	1.8 (0.6)	2.3 (0.6)	1.6 (0.5)	0.10
LED (mg)	167.9 (55.8)	158.3 (72.2)	170.5 (54.6)	0.87
LEDD (mg)	780.6 (376.8)	616.7 (325.3)	825.3 (391.3)	0.63

^a^ n, Mean (SD).

^b^ Wilcoxon rank sum test.

MSA, Multiple system atrophy; PD, Parkinson’s disease; OH, Orthostatic hypotension; MDS-UPDRS III, Part 3 of Movement Disorder Society Unified Parkinson’s Disease Rating Scale; LED, Levodopa equivalent dose; LEDD, Levodopa equivalent daily dose.

### 3.2 Levodopa-induced changes in cardiovascular autonomic physiomarkers

**FIGURE 4:**
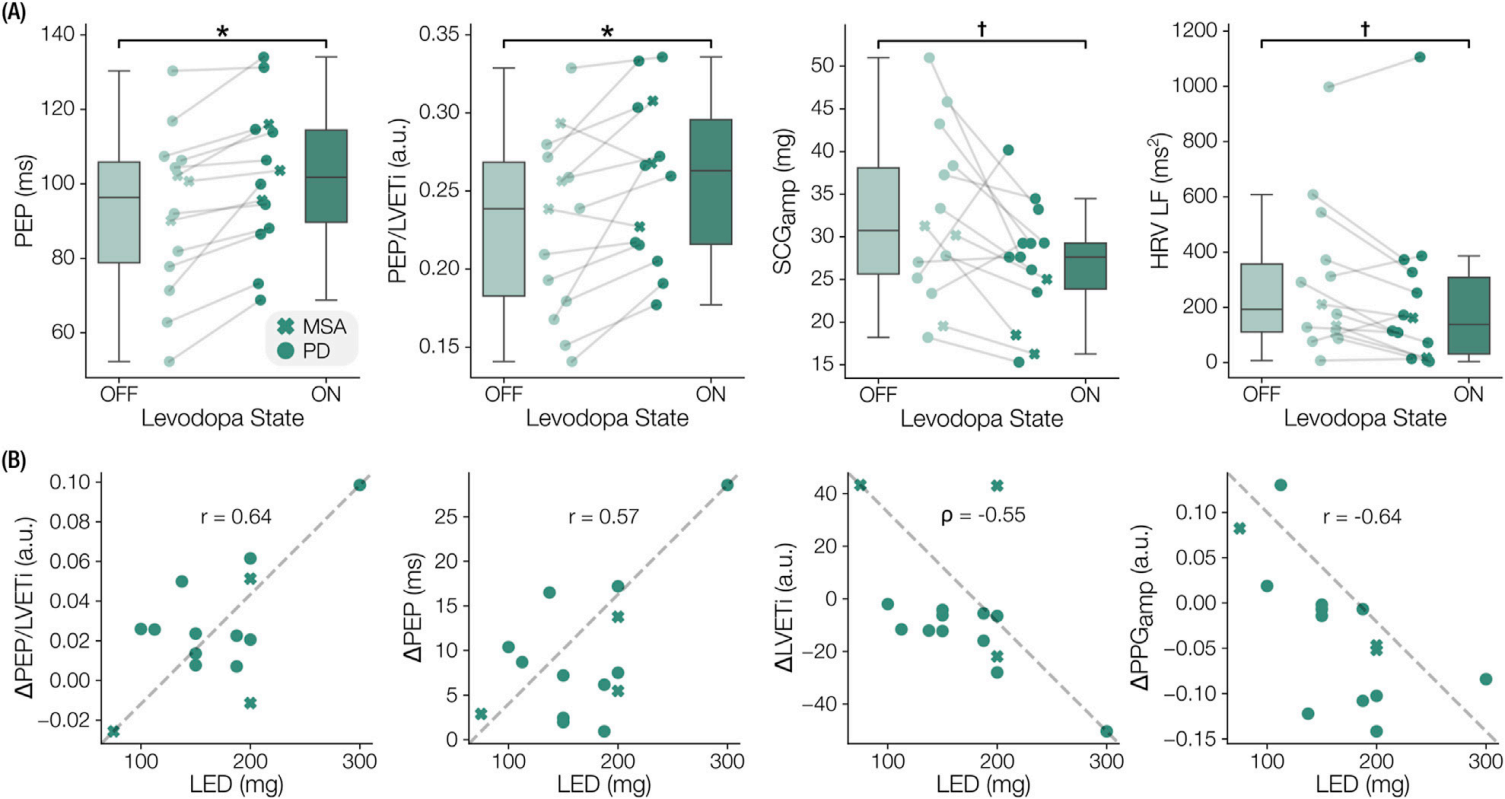
Notable levodopa-induced physiomarker changes and dose-response correlations. **(A)** Boxplots indicating significant physiomarkers changes from prior to levodopa (OFF) to an hour after (ON). Pre-ejection period (PEP), the ratio of PEP to left ventricular ejection time index (PEP/LVETi), seismocardiogram amplitude ( ${\text{SCG}}_{\text{amp}}$ ), and the low frequency band of heart rate variability (HRV-LF) are shown. **(B)** Correlation plots between the administered levodopa equivalent dosage (LED) and the magnitude of OFF-ON physiomarker changes in PEP/LVETi, PEP, LVETi, and photoplethysmogram amplitude ( ${\text{PPG}}_{\text{amp}}$ ) are depicted. Pearson’s r and Spearman’s ρ were used, depending on the distribution of the data. * $p_{\text{adj}}\;<$ 0.05; † p < 0.05.

Several seated physiomarkers differed significantly between the OFF and ON states measured in clinic, as depicted by the boxplots in Figure 4A. Specifically, significant increases in SCG-derived PEP ( ${\text{p}}_{\text{adj}}$ = 0.003, t = −4.560, d = 1.264) and PEP/LVETi ( ${\text{p}}_{\text{adj}}$ = 0.025, t = −3.175, d = 0.880) were observed. Decreases in ${\text{SCG}}_{\text{amp}}$ (p = 0.047, t = 2.186, d = 0.606) and HRV-LF (p = 0.040, t = 2.269, d = 0.629) were also found, though not significant after multiple comparison correction. For the N = 36 levodopa responses captured at home, there were no significant physiomarker changes from OFF to ON states. The results of all clinical and at-home OFF-ON comparisons are recorded in Supplementary Tables IV, V, respectively.

### 3.3 Relationship of physiomarker changes to levodopa dosage

We observed that the magnitudes of some physiomarker responses were associated with the LED administered as part of the clinical protocol. SCG-based features such as the change in PEP (p = 0.031, r = 0.573), LVETi (p = 0.042, ρ = -0.547), and PEP/LVETi (p = 0.014, r = 0.635) demonstrated noteworthy correlations. Similarly, the change in ${\text{PPG}}_{\text{amp}}$ (p = 0.012, r = −0.644) correlated to the LED. While these correlations did not survive multiple comparison correction, and should be cautiously interpreted, they are near the threshold for significance and may warrant further investigation. The statistics associated with all physiomarker correlations to LED are displayed in Supplementary Table VI.

### 3.4 Levodopa response differences between OH and no-OH groups

**FIGURE 5:**
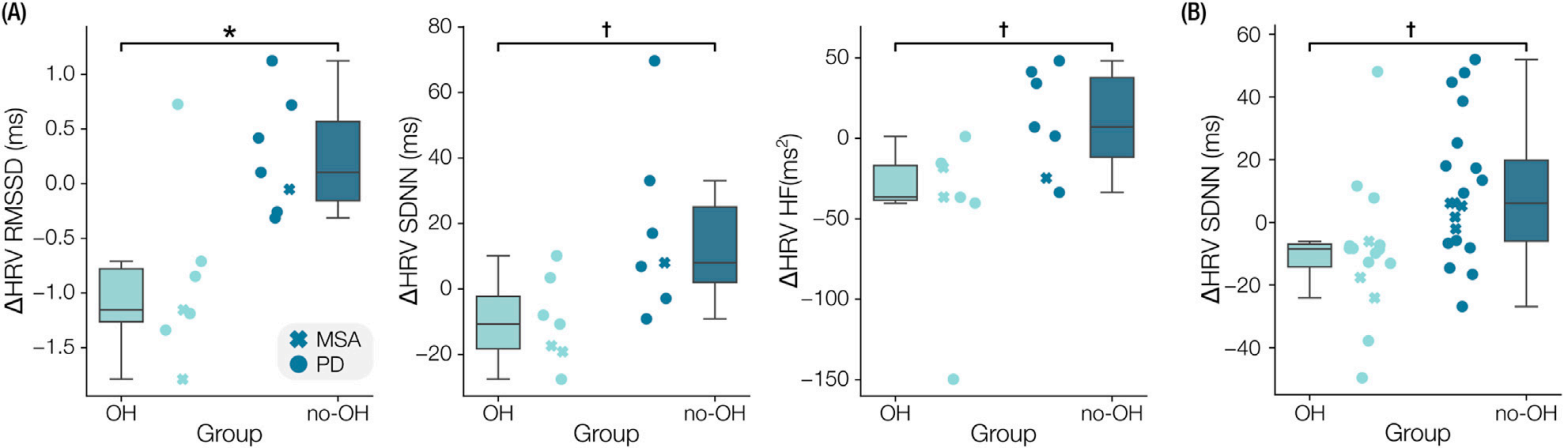
Comparison of levodopa responses between participants with orthostatic hypotension (OH) and without OH (no-OH). **(A)** Boxplots of the clinical levodopa responses are shown for the root-mean-square-of-successive-differences (RMSSD), standard deviation of normal-normal intervals (SDNN), and high frequency of heart rate variability (HRV-HF). **(B)** Boxplot of the difference in SDNN measured in the at-home responses. * $p_{\text{adj}}\;<$ 0.05; † p < 0.05.

In participants with OH, the in-clinic RMSSD of NN intervals was significantly reduced ( ${\text{p}}_{\text{adj}}$ = 0.048, t = −3.171, d = 1.831) relative to the no-OH group following levodopa. Additionally, SDNN (p = 0.031, t = −2.433, d = 1.404) and HRV-HF (p = 0.017, U = 6.0, d = 1.362) were reduced, nonsignificant after correction, in the OH group relative to the no-OH group. In the at-home responses, ON-state SDNN was reduced (p = 0.037, t (34) = -2.081), which was nonsignificant after adjustment, in the OH group compared to the no-OH group for the n = 12 participants with at-home levodopa responses. Boxplots are shown for the discussed comparisons in Figure 5, and the results of all statistical tests are summarized in Supplementary Tables VII, VIII.

### 3.5 Case study: physiomarker time-series during adverse levodopa response

**FIGURE 6:**
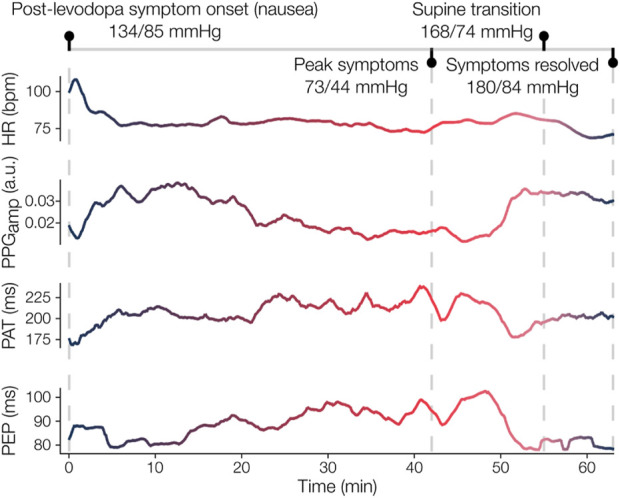
Case study of adverse levodopa reaction in participant with PD and OH. The participant initially experienced nausea about 40 min after taking levodopa, which worsened over the next 40 min. During this time, a drop in blood pressure (BP) from 134/85 mmHg (systolic/diastolic) to 73/44 mmHg was recorded. Concurrently, the wearable patch measured an increase in pulse arrival time (PAT) and pre-ejection period (PEP), which similarly indicates decreased arterial stiffness and decreased cardiac contractility given the stable heart rate (HR). After lying supine, the participant’s BP eventually surged (180/84 mmHg) and their symptoms dissipated. The marked responses in PAT and PEP, as well as the increase in photoplethysmogram amplitude ( ${\text{PPG}}_{\text{amp}}$ ), upon assuming a supine position further illustrate the return to a more normal physiological state.

A participant with PD and OH experienced an adverse reaction to levodopa prior to completion of the clinical protocol. Symptoms of nausea precipitated a marked fall in BP (SBP/DBP: 73/44 mmHg) roughly 80 min after levodopa administration. The participant recovered by lying supine until BP increased to 168/74 mmHg and the feelings of nausea were reduced. Time-series of exemplary physiomarkers from onset of nausea, about 40 min following administration of 300 mg LED, to the resolution of symptoms are shown in Figure 6. The physiomarkers depict the underlying hemodynamic changes throughout the symptomatic period and subsequent recovery, thus illustrating the sensitivity of the measured waveforms to CVA dysfunction in an individual participant.

## 4 Discussion

### 4.1 Principal findings

In this work, we demonstrate a novel wearable-based approach for evaluating the CVA dynamics associated with levodopa in patients with parkinsonism. Notably, this study is the first to employ a small form-factor chest patch that captures multiple physiological paradigms—namely the electrical and cardiomechanical activity of the heart alongside vascular reactivity—to extract meaningful measures of CVA state related to the effects of levodopa. We found that markers of cardiac contractility obtained from SCG waveforms were sensitive to the administration of levodopa and depicted a potential negative inotropic effect. We further explored the OFF-ON transitions and observed relationships between physiomarker changes and levodopa dosages as well as differences in HRV responses between participants with and without OH. Finally, we illustrated the utility of continuous monitoring via the wearable patch by capturing levodopa responses in an uncontrolled, at-home environment where the CVA effects of levodopa appeared subtler. The presented results demonstrate the feasibility of wearable sensing for improved monitoring of CVA function as well as grant further insight into the influence of levodopa on CVA health in patients with parkinsonism.

The multimodal sensing approach employed in this study enabled a more comprehensive assessment of CVA function than was captured in several prior studies examining the effects of levodopa, which often measure HRV alone (Sriranjini et al., 2011; Ruonala et al., 2015). The extracted physiomarkers themselves proved to be sensitive to the levodopa-induced changes in CVA state, primarily in the clinical data, during both periods of rest (Figures 4, 5) and the exemplary symptomatic period (Figure 6). In addition to a richer set of physiomarkers, the form factor of the wearable patch and acquisition of ECG, SCG, and PPG signals also facilitated remote continuous monitoring of ambulatory hemodynamic responses to levodopa, which have not been previously reported. The physiomarkers relying on cross-modal information, specifically the systolic timing interval measures, were key to characterizing CVA changes. Further work on coupling the presented physiomarkers with the accelerometer’s measures, which provide activity and postural context (Godfrey et al., 2011) and crude indices of motor symptoms in parkinsonism (Thorp et al., 2018), could lead to a more holistic solution for monitoring aspects of both non-motor and motor effects of levodopa.

Our results provide further support for the previously reported negative inotropic effect associated with levodopa in patients with α SNA (Wolf et al., 2006; Noack et al., 2014). The significant increases in PEP and PEP/LVETi with concurrent—though statistically nonsignificant—decreases in LVETi and ${\text{SCG}}_{\text{amp}}$ following oral levodopa administration, are indicative of decreased cardiac contractility (Ahmed et al., 1972) or reduced beta-adrenergic sympathetic activity (Kelsey, 2012). When additionally considering the lack of notable changes to HR and in physiomarkers related to arterial stiffness, such as PTT or ${\text{PPG}}_{\text{amp}}$ (Reisner et al., 2008; Mukkamala et al., 2015), these findings align with the theory that a potentially hypotensive effect of levodopa involves cardioinhibitory effects, rather than solely direct vasomotor mechanisms (Noack et al., 2014). We also observed that HRV parameters were largely unaltered except for a significant, before correction, reduction in HRV LF. As reported HRV responses to levodopa are widely varied, with prior work showing increases (Ludwig et al., 2007; Sriranjini et al., 2011), decreases (Ruonala et al., 2015), or no change (Sénard et al., 1995; Noack et al., 2014) in HRV parameters, further study is needed to characterize levodopa’s effects on HRV.

Whereas the levodopa evoked notable changes in physiomarkers measured in the clinic, there were no similar findings in the at-home data outside of the reduced RMSSD in the OH group compared to the no-OH group. Several factors could be influencing this finding. Given the at least 12-h washout period preceding the clinical protocol, the CVA effects of levodopa measured in clinic may have been exaggerated compared to typical administrations. Potential habituation to levodopa may have contributed to diminished autonomic responses measured at home, where levodopa was taken more regularly by participants. Inaccuracies in the patient-reported administration times could have introduced differences in the timing of the OFF-ON periods as defined for the in-clinic portion. Though the analyzed OFF-ON periods at home were selected similarly to the clinical periods, i.e., same posture with minimal activity, the lack of control in the at-home environment likely introduced more noise to the physiomarker extraction pipeline, thereby reducing the comparability between the clinical and home data. Finally, the effects of levodopa could have been obscured due to participants with OH taking other pressor agents concurrent with levodopa in order to reduce its potential hypotensive side effects. As this is the first study, to the best of our knowledge, to characterize at-home CVA responses to levodopa in this manner, future studies are warranted to validate these findings and address potential confounding factors, such as concurrent medication use, variable dosing schedules, and the influence of environmental and activity-related noise on the measurement of wearable physiomarkers.

Many studies examining the CVA effects of levodopa administer a fixed LED for all participants (Sriranjini et al., 2011; Noack et al., 2014). As the participants in this study took their typical LED, we were able to explore several significant, before correction, correlations between the magnitude of the physiomarkers changes and the participant’s LED. These correlations suggest that greater doses elicit larger changes in SCG-based systolic timing intervals, trending towards the reduced contractile state as discussed earlier. The relationship to ${\text{PPG}}_{\text{amp}}$ would additionally indicate a greater level of vasoactivity with increased LED. We hypothesize that this reduction in ${\text{PPG}}_{\text{amp}}$ is associated with a reduction in pulse pressure, which could be dictated by decreases in stroke volume (Reisner et al., 2008; Berkebile et al., 2023), rather than sympathetically-mediated vasoconstriction given the known post-ganglionic sympathetic denervation in PD (Jain and Goldstein, 2012). While these findings did not retain significance after correction, validation in larger cohorts will reveal the association between LED and the reported physiomarker changes. If upheld, these findings could improve individualized dosing of levodopa, particularly for people suffering from OH.

In analyzing how levodopa responses vary between people with and without CVA dysfunction, specifically OH, we found that HRV parameters responded distinctly between groups. A significant reduction in RMSSD was accompanied by significant, before correction, reductions in SDNN and HRV HF in the OH group relative to the no-OH group following levodopa administration in the clinic. The at-home data exhibited similar magnitudes of HRV changes in the OH and no-OH groups compared to the clinical data, with reduced HRV in the OH group relative to the no-OH group. The HRV reduction following levodopa in the OH group may indicate an exacerbation of baroreflex dysfunction (Earl et al., 2024), which results in impaired heart rate and peripheral vasoconstriction responses (Kaufmann and Palma, 2017). These results, though exploratory, suggest that levodopa may further impair autonomic regulation mostly in patients with known or latent CVA dysfunction (Cani et al., 2024), highlighting the need for improved monitoring of CVA function when initiating or escalating an individual’s dose of LED.

### 4.2 Limitations

This study has several limitations that should be considered when interpreting the results. Firstly, the number of participants and inclusion of multiple pathologies (PD and MSA) constrains the inferences made in this work. Specifically, CVA effects of levodopa are more commonly examined in PD and not MSA (Sénard et al., 1995; Sriranjini et al., 2011; Noack et al., 2014). Indeed, peripheral cardiac sympathetic denervation is near universal in PD (Goldstein, 2003), as opposed to severe central autonomic neurodegeneration in MSA, which could impact cardiac contractility findings here and in prior work (Noack et al., 2014). Yet, comparisons of PEP, PEP/LVET, and LVET were significant participants with PD alone Additionally, the complexity of the statistical modeling was limited by the number of subjects. This limitation may have masked subtler effects of levodopa on CVA function particularly for ambulatory analyses. Furthermore, there was no direct comparison with gold-standard measurements of cardiomechanical activity; however, the observed levodopa-induced changes in CVA function align with prior findings using controlled protocols and bench-top equipment (Noack et al., 2014; Ruonala et al., 2015; Wolf et al., 2006). Additionally, the ambulatory portion of this study was completely uncontrolled, which introduces variability to the analyses of levodopa responses. While the participant diaries provided some insight, a participant’s autonomic state may have been influenced by other activities, such as eating, or mental state (e.g., stress). The repeatability of at-home responses could be better assessed in future work with a semi-controlled measure at home whereby a participant performs a pre-and-post levodopa supine-to-stand or sit-to-stand test.

Future work should aim to better understand the clinical implications and mechanisms of the observed effects of levodopa, both in the lab and at home. In larger populations, exploring additional factors and interactions might unveil, or refute, further relationships between levodopa, CVA dysfunction, concomitant pressor agents, and parkinsonian state. Specifically, assessing the interaction between levodopa state (OFF/ON) and orthostatic responses with the wearable patch could provide insight into levodopa’s influences on cardiomechanical activity during postural changes. The integration of wearable sensing and clinical decision-making is promising but remains unproven in individuals with α SNA. Future work should determine if remote monitoring can improve both motor and non-motor outcomes by optimizing and personalizing levodopa dosing strategies.

## Data Availability

The raw data supporting the conclusions of this article will be made available by the authors, without undue reservation.
