# Supplementary material for: Wearable multimodal sensing for quantifying the cardiovascular autonomic effects of levodopa in parkinsonism
Source: Front Netw Physiol. 2025 Apr 24;5:1543838. doi: 10.3389/fnetp.2025.1543838 (PMC12058781; doi:10.3389/fnetp.2025.1543838)
Supplement: Supplementary file 1 [file DataSheet1.pdf]

Supplementary Material

1 SUPPLEMENTARY TABLES

Table I. Heart rate and blood pressure during clinical protocol

Parameter	no-OH N = 7[1]	OH N = 7[1]	PD N = 11[1]	MSA N = 3[1]
**HR (bpm)**				
Seated	63.17 (12.87)	72.95 (19.10)	67.08 (18.39)	71.67 (6.55)
Supine	59.71 (10.51)	67.94 (14.34)	62.43 (14.09)	68.96 (4.80)
Standing	68.84 (12.11)	82.13 (22.67)	73.70 (20.36)	82.06 (11.69)
**SBP (mmHg)**				
Seated	147.96 (17.63)	138.59 (36.11)	149.71 (27.37)	119.70 (13.89)
Supine	147.56 (17.47)	162.79 (38.26)	158.48 (32.05)	143.06 (17.25)
Standing	157.90 (24.11)	120.77 (28.11)	146.73 (27.27)	112.22 (37.46)
**DBP (mmHg)**				
Seated	78.84 (12.66)	78.93 (14.47)	81.53 (12.60)	69.20 (11.72)
Supine	77.90 (12.21)	85.87 (14.27)	83.37 (13.25)	76.44 (15.36)
Standing	85.53 (10.20)	76.09 (14.05)	84.74 (10.47)	66.39 (10.73)

[1]n; Mean (SD)
Parkinson's disease (PD); Multiple system atrophy (MSA); Orthostatic hypotension (OH); Heart rate (HR); Systolic blood pressure (SBP); Diastolic blood pressure (DBP)

Table II. Motor Testing in OFF and ON States

Parameter	OFF-Mean	OFF-SD	ON-Mean	ON-SD	Test	Statistic	Cohen's d	p
MDS-UPDRS III	31.857	9.840	20.142	8.872	t-test	7.978	2.212	<0.001
Hoehn & Yahr	2.142	0.515	1.785	0.557	Wilcoxon	0.000	0.745	0.025

Table III. Concomitant blood pressure-related medications in participants on levodopa

Participant Group	OH	Medication	Dosage
MSA	y	Midodrine	2.5 mg qD
PD	y	Olmesartan	10 mg qD
PD	y	Midodrine	10 mg tid
PD	y	Midodrine	10 mg p.r.n
MSA	y	Midodrine	7.5 mg tid
PD	n	Amlodipine	7.5 mg qD

Table IV. Clinical Levodopa OFF-ON Comparisons

Feature	OFF-Mean	OFF-SD	ON-Mean	ON-SD	Test	Statistic	Cohen's d	p	p_{adj}
HR	70.969	13.714	73.087	13.385	t-test	-1.342	0.372	0.202	0.404
PPG_{amp}	0.258	0.189	0.226	0.179	t-test	1.562	0.433	0.142	0.165
PAT	189.356	39.755	199.479	34.005	t-test	-1.880	0.521	0.082	0.126
PTT	96.804	39.380	97.478	33.438	t-test	-0.126	0.035	0.901	0.901
SCG_{amp}	32.251	9.488	26.829	6.682	t-test	2.186	0.606	0.047	0.111
PEP	92.610	20.730	101.873	18.643	t-test	-4.560	1.264	0.000	0.003
LVETi	406.658	28.312	400.216	23.029	Wilcoxon	25.0	0.273	0.090	0.126
PEP/LVETi	0.229	0.054	0.255	0.049	t-test	-3.175	0.880	0.007	0.025
HRV-LF	289.273	259.854	222.872	276.994	t-test	2.269	0.629	0.040	0.245
HRV-HF	93.947	84.557	78.168	90.999	Wilcoxon	39.0	0.336	0.426	0.639
HRV-LF/HF	3.796	1.503	3.788	1.830	t-test	0.012	0.003	0.989	0.989
HRV-SDNN	36.212	17.033	40.027	34.360	t-test	-0.576	0.160	0.573	0.688
HRV-RMSSD	5.314	1.271	4.989	1.728	t-test	1.378	0.382	0.191	0.404

Table V. At-home Levodopa OFF-ON Comparisons

Feature	Estimate	Conf. lower	Conf. upper	SE	DF	t	p	p_{adj}
HR	-0.294	-3.192	2.604	1.478	70	-0.199	0.842	0.921
PPG_{amp}	0.008	-0.079	0.096	0.044	70	0.187	0.851	0.851
PAT	2.613	-10.627	15.854	6.755	70	0.386	0.698	0.851
PTT	2.114	-10.064	14.292	6.213	70	0.340	0.733	0.851
SCG_{amp}	0.002	-0.002	0.006	0.002	70	0.824	0.409	0.851
PEP	0.682	-5.905	7.271	3.361	70	0.203	0.839	0.851
LVETi	-5.483	-17.476	6.510	6.119	70	-0.896	0.370	0.851
PEP/LVETi	0.005	-0.016	0.027	0.011	70	0.516	0.605	0.851
HRV-LF	45.687	-45.424	136.798	46.486	70	0.982	0.325	0.921
HRV-HF	2.408	-45.678	50.495	24.534	70	0.098	0.921	0.921
HRV-LF/HF	0.5	-0.321	1.321	0.419	70	1.192	0.233	0.921
HRV-SDNN	1.718	-6.406	9.843	4.145	70	0.414	0.678	0.921
HRV-RMSSD	-0.166	-0.763	0.430	0.304	70	-0.547	0.584	0.921

Table VI. Dose-Response Correlation

Feature	Correlation	Coefficient	p	p_{adj}
HR	Pearson	-0.269	0.351	0.858
PPG_{amp}	Pearson	-0.644	0.012	0.051
PAT	Pearson	0.358	0.208	0.292
PTT	Pearson	0.146	0.617	0.617
SCG_{amp}	Pearson	-0.241	0.405	0.472
PEP	Pearson	0.573	0.031	0.074
LVETi	Spearman	-0.547	0.042	0.074
PEP/LVETi	Pearson	0.635	0.014	0.051
HRV-LF	Pearson	0.165	0.572	0.858
HRV-HF	Spearman	0.185	0.252	0.858
HRV-LF/HF	Pearson	-0.420	0.134	0.807
HRV-SDNN	Pearson	0.003	0.990	0.990
HRV-RMSSD	Pearson	0.030	0.918	0.990

Table VII. Comparison of Clinical Levodopa Responses between OH and no-OH Groups

Feature	OH-Mean	OH-SD	no-OH-Mean	no-OH-SD	Test	Statistic	Cohen's d	p	p_{adj}
HR	4.745	4.639	-0.507	5.425	t-test	1.802	1.040	0.096	0.144
PPG_{amp}	-0.027	0.067	-0.037	0.081	t-test	0.249	0.143	0.807	0.807
PAT	6.947	23.156	13.296	14.048	MWU	19.0	0.331	0.534	0.807
PTT	-3.338	23.095	4.685	13.134	t-test	-0.739	0.427	0.473	0.807
SCG_{amp}	-7.301	8.716	-3.542	8.766	t-test	-0.744	0.430	0.470	0.807
PEP	10.177	8.497	8.349	5.777	t-test	0.435	0.251	0.670	0.807
LVETi	-7.042	25.805	-5.842	21.026	MWU	29.0	0.050	0.620	0.807
PEP/LVETi	0.028	0.035	0.024	0.023	t-test	0.262	0.151	0.797	0.807
HRV-LF	-112.085	66.325	-20.717	116.973	t-test	-1.664	0.960	0.121	0.146
HRV-HF	-42.153	45.967	10.594	29.777	MWU	6.0	1.362	0.017	0.052
HRV-LF/HF	0.342	1.881	-0.359	2.658	MWU	33.0	0.304	0.317	0.317
HRV-SDNN	-9.887	12.166	17.516	24.759	t-test	-2.433	1.404	0.031	0.063
HRV-RMSSD	-0.899	0.738	0.249	0.492	t-test	-3.171	1.831	0.008	0.048

Table VIII. Comparison of At-home Levodopa Responses between OH and no-OH Groups

Feature	Estimate	Conf. lower	Conf. upper	SE	DF	t	p	p_{adj}
HR	4.192	-14.694	23.079	9.636	34	0.435	0.663	0.796
PPG_{amp}	-0.017	-0.131	0.095	0.057	34	-0.308	0.757	0.888
PAT	-7.360	-32.567	17.847	12.861	34	-0.572	0.567	0.888
PTT	-5.134	-29.665	19.396	12.515	34	-0.410	0.681	0.888
SCG_{amp}	0.001	-0.005	0.008	0.003	34	0.453	0.650	0.888
PEP	-1.169	-10.376	8.037	4.697	34	-0.249	0.803	0.888
LVETi	6.965	-11.382	25.314	9.361	34	0.744	0.456	0.888
PEP/LVETi	-0.002	-0.031	0.027	0.015	34	-0.140	0.888	0.888
HRV-LF	-175.472	-376.873	25.927	102.757	34	-1.707	0.087	0.263
HRV-HF	-59.890	-156.510	36.730	49.297	34	-1.214	0.224	0.448
HRV-LF/HF	-0.117	-1.683	1.449	0.799	34	-0.146	0.883	0.883
HRV-SDNN	-18.280	-35.494	-1.067	8.782	34	-2.081	0.037	0.224
HRV-RMSSD	-0.493	-1.598	0.611	0.563	34	-0.875	0.381	0.571
